# Influence of Aggregate Coated with Modified Sulfur on the Properties of Cement Concrete

**DOI:** 10.3390/ma7064739

**Published:** 2014-06-20

**Authors:** Swoo-Heon Lee, Ki-Nam Hong, Jae-Kyu Park, Jung Ko

**Affiliations:** 1 Department of Civil Engineering, University of Texas at Arlington, Arlington, TX 76019, USA; E-Mail: swooheon@uta.edu; 2 School of Civil Engineering, Chungbuk National University, Chungbuk 361-763, Korea; E-Mail: kaga0618@hanmail.net; 3 Railway Inspection Section, Korea Infrastructure Safety & Technology Corporation, Gyeonggi 411-758, Korea; E-Mail: kjungk33@hanmail.net

**Keywords:** modified sulfur, coated aggregate, durability, strength, freezing-thawing, acid resistance

## Abstract

This paper proposes the mixing design of concrete having modified sulfur-coated aggregate (MSCA) to enhance the durability of Portland cement concrete. The mechanical properties and durability of the proposed MSCA concrete were evaluated experimentally. Melting-modified sulfur was mixed with aggregate in order to coat the aggregate surface at a speed of 20 rpm for 120 s. The MSCA with modified sulfur corresponding to 5% of the cement weight did not significantly affect the flexural strength in a prism concrete beam specimen, regardless of the water-cement ratio (W/C). However, a dosage of more than 7.5% decreased the flexural strength. On the other hand, the MSCA considerably improved the resistance to the sulfuric acid and the freezing-thawing, regardless of the sulfur dosage in the MSCA. The coating modified sulfur of 5% dosage consequently led to good results for the mechanical properties and durability of MSCA concrete.

## 1. Introduction

Reinforced concrete has been widely used as construction material due to its versatility. However, several factors can cause the deterioration of concrete structures. Currently, abnormal climate and air pollution have accelerated the deterioration of concrete structures. The main factors behind the deterioration of concrete are considered to be the salt damage, carbonation, chemical erosion, frost damage, and drying shrinkage. The deterioration leads to steel corrosion and sectional-area reduction. Thus, such damage can cause the collapse of structures because it can result in strength loss and unsafe conditions. Neville explained the mechanisms of concrete deterioration [1]: (1) Carbonation—concrete carbonation results when CO_2_ reacts with Ca(OH)_2_. Carbonation starts on the concrete surface and moves slowly to the interior of the concrete because CO_2_ in the air penetrates to the interior of the concrete through capillarity in the cement paste; (2) Corrosion—steel reinforcements can be destroyed by chloride ions that penetrate the concrete; (3) Freeze and thaw cycles—because concrete is porous, water is trapped and absorbed in the pores. The increased volume of water can lead to cracking when the water in concrete freezes; (4) Sulfate attack—the soluble sulfates in groundwater may react with a hydrate from cement. Wang *et al.* [2,3,4,5] recently carried out a research project titled “Analysis of Climate Change Impacts on the Deterioration of Concrete Infrastructure”, in Australia. The report included (i) a review of the mechanisms, practice, modeling, and simulations of concrete deterioration; (ii) modeling and simulations of concrete deterioration processes and adaptation options; and (iii) case studies of concrete deterioration and adaption. Although the extent of damage due to deterioration differs from country to country because the climate and environmental conditions vary, research to prevent the deterioration of concrete has become a great shared interest. Particularly, research on how to integrate industrial waste into the concrete mix has been conducted to improve the durability of concrete [6,7,8,9].

Attempts to use sulfur as a construction material began around 1921 to consume the surplus sulfur from a mine named Big Doom in Texas, USA. Bacon and Davis [10] found that the mixture of 60% sand and 40% sulfur had high strength and acid resistance. However, the sulfur concrete had problems of flexural strength reduction, cubic expansion, and micro-crack occurrence due to repeated temperature changes. To overcome these problems, studies on the modification of sulfur have been carried out by many researchers [11,12,13]. Their sulfur modifier systems were a mixture of dicyclopentadiene and oligomers of cyclopentadiene or dicyclopentadiene, and the melting temperatures of their modified sulfur were in the range of 118–140 °C. Sulfur concrete has several advantages over Portland cement concrete [14,15,16,17,18]: (1) its resistance to broadly-based acid and salinity is high; (2) the required mechanical property can be manifested in 24 h due to its fast hardening; (3) it has high tensile, compressive, and flexural strength as well as high fatigue resistance; (4) it can be manufactured all-year-round or even in subfreezing weather; (5) it can be used as a waterproof material due to the hydrophobicity of sulfur; and (6) it has always been recyclable. Thus, sulfur concrete can be used as a replacement material for Portland cement not only in places where acid and salinity can cause damage but also in places with extreme climate where freezing-thawing can repeatedly occur [16].

Recently, studies to improve the mechanical properties and durability of concrete by using a coating aggregate have also been conducted. Li *et al.* [19] found that a coating recycled aggregate with pozzolanic powder improved the concrete slump, compressive strength, flexural strength, and precision in the interfacial transition zone (ITZ) compared to Portland cement concrete. Kong *et al.* [20] proposed a triple mixing method (TM) to realize surface-coating aggregate using pozzolanic materials. This method enhanced the strength and durability of recycled aggregate concrete more than when a double mixing method (DM) was used. Compared to concrete with dispersed latex polymer in the bulk past, the fracture energy was increased in the concrete with a latex polymer-coated aggregate by Morin *et al.* [21]. The compressive strength in the pre-coating recycled aggregate concrete was increased by 10% with an increased water-cement ratio in comparison with the control concrete. The coating was found to reduce the strength loss of mortar through a sulfate attack test conducted by Zhihui *et al.* [22].

To use sulfur as a construction material, it is necessary to heat the aggregate and filler as well as to melt the modified sulfur at high temperature [16]. The heating cost has been an impediment to the application of sulfur in large-scale construction sites. In this study, a modified sulfur-coated aggregate (MSCA) concrete was designed to integrate the acid resistance and freezing-thawing resistance of sulfur into Portland cement concrete. The performance of the MSCA concrete was evaluated experimentally.

## 2. Test Results and Discussion

### 2.1. Properties of Hardened Concrete

The water absorptions of R40-CON, R40-NS5, R40-NS7.5, and R40-NS10 were 3.38%, 4.17%, 4.18%, and 4.20%, respectively. And the total porosities of corresponding admixtures were 7.74%, 9.48%, 9.67%, and 10.06%, severally. It was confirmed, from test results, that the MSCA increased both absorption and porosity of concrete, but the modified sulfur content at the coated aggregates slightly affected them. This result can be explained as followings: (1) the coated aggregate with modified sulfur causes its absorption to be reduced; (2) the space occupied by surplus water forms the high porosity in the MSCA concrete. The bulk densities of R40-CON, R40-NS5, R40-NS7.5, and R40-NS10 were 2.29, 2.26, 2.26, and 2.25 Mg/m^3^, respectively. Those were not much different regardless of amount of modified sulfur.

### 2.2. Compressive Strength

**Figure 1:**
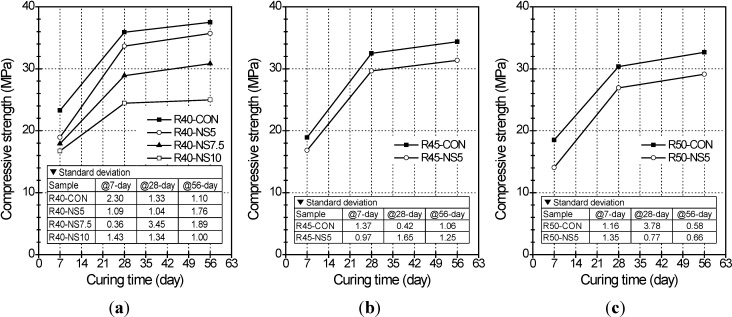
Compressive strength of cylinders with (**a**) water-cement ratio (W/C) of 40%; (**b**) W/C of 45%; and (**c**) W/C of 50% with respect to W/C and curing time.

Figure 1 shows the compressive strength changes in the concrete cylinders with MSCAs, which were coated with various amounts of sulfur, in relation to W/C and curing time. The compressive strengths shown in Figure 1 are the average values of three specimens for each mixture. For admixtures with W/C ratio of 40%, the compressive strengths of R40-NS5, R40-NS7.5 and R40-NS10 at a 7-day age had decreased by 19%, 23% and 28% relative to R40-CON, respectively (see Figure 1a). At a 28-day age, the compressive strength of corresponding admixtures decreased by 6%, 20%, and 32%. At 56 days, these values had decreased to 5%, 18%, and 33%. The reduction rate of compressive strength of R40-NS5 decreased with passing curing age, whereas those of R40-N7.5 and R40-N10 were almost constant regardless of curing age. It was obvious from test results that the high dosage of modified sulfur may cause the cement setting problem and thus could delay the hydration process in concrete.

The compressive strength of the MSCA concrete decreased further when a higher amount of modified sulfur was used to coat the aggregate. There could be three reasons: (1) the remains of coated sulfur can act as impurities or light-weight aggregate in the concrete; (2) the additional quantity of superplasticizer needed to obtain an identical slump in concrete with more sulfur can lead to the formation of more pores in the cement paste; (3) the more amounts of modified sulfur owing to its low stiffness and strength result in the weaker microstructure of ITZ, leading to a decreased compressive strength.

Of the specimens with 5% MSCA, the compressive strengths at 56 days in R40-NS5, R45-NS5, and R50-NS5 cylinder samples (see Figure 1b,c) had decreased by 5%, 9%, and 11% from that of each control sample with normal aggregate. In other words, the compressive strength tended to decrease with an increased W/C. A possible explanation is that the surplus of modified sulfur may have a greater effect on the strength of concrete with a higher W/C because the amount of cement decreased with the higher W/C.

### 2.3. Flexural Strength

**Figure 2:**
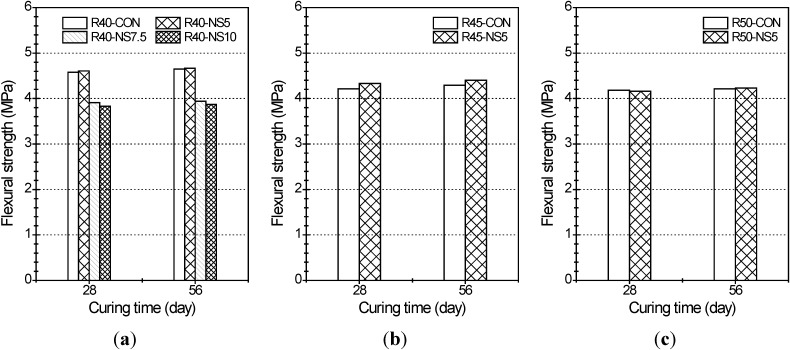
Flexural strength of prism beams with (**a**) W/C of 40%; (**b**) W/C of 45%; and (**c**) W/C of 50% in relation to W/C and curing time.

The flexural strengths of the prism beams are plotted in Figure 2. The strengths shown in Figure 2 are the averaged values of three specimens for each mixture. The flexural strengths at 28 and 56 days were almost the same for each W/C. Of the specimens with a W/C of 40% (see Figure 2a), the strength at 28 days of R40-NS5 had increased by about 0.7% as compared to the control specimen of R40-CON. On the contrary, the strengths at 28 days of the others had decreased up to 15%–16%. The results of this experiment are in agreement with the experimental result obtained in a study by Morin *et al.* [21], which used latex-coated aggregate. The flexural strength loss in the concrete with aggregate coated by more than 7.5% of modified sulfur may have the same explanation as the loss of compressive strength discussed above.

Additionally, the flexural strengths at 56 days of specimens with different W/C and 5% MSCA had increased when compared with each control sample, but the variation rate was slight (e.g., 0.4% for W/C = 40%, 2.6% for W/C = 45% and 0.5% for W/C = 50%). In other words, the 5% MSCA did not significantly affect the flexural strength loss regardless of W/C.

### 2.4. Length Change

**Figure 3:**
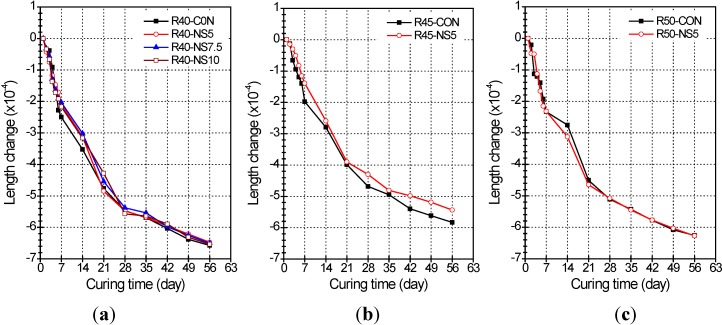
Length change of prism beams with (**a**) W/C of 40%; (**b**) W/C of 45%; and (**c**) W/C of 50% in relation to W/C and curing time (unit: mm/mm).

The length change of concrete with passing time is presented in Figure 3. The averaged length changes of R40-CON, R40-N45, R40-N7.5, and R40-N10 admixtures at 7-day age were −2.50 × 10^−4^, −2.10 × 10^−4^, −2.03 × 10^−4^, and −2.17 × 10^−4^ mm/mm, repetitively (see Figure 3a). The MSCA led to a slight decrease of length change, but the amount of modified sulfur at the MSCA did not significantly affect the length change. At the age of 56 days, the length changes of corresponding admixtures had increased by −6.57 × 10^−4^, −6.47 × 10^−4^, −6.50 × 10^−4^, and −6.53 × 10^−4^ mm/mm, respectively (see Figure 3a). The length change of MSCA concrete increased with the higher content of modified sulfur, but it was determined that the modified sulfur had little effect on the length changes because the change rates of MACA concrete were almost similar to that of R40-CON specimen. The length changes at 7–56 days in specimens having a W/C of 45% (see Figure 3b) were greater in each control sample than in the specimen with the MSCA. On the other hand, the length changes of two specimens with a 50% W/C (see Figure 3c) went up and down until 28 days. After 28 days of curing, the length changes of two specimens were almost identical. Based on the length change observations, it was found that the length change due to the MSCA was slightly smaller than or equal to that of the control specimens.

### 2.5. Freezing and Thawing

**Figure 4:**
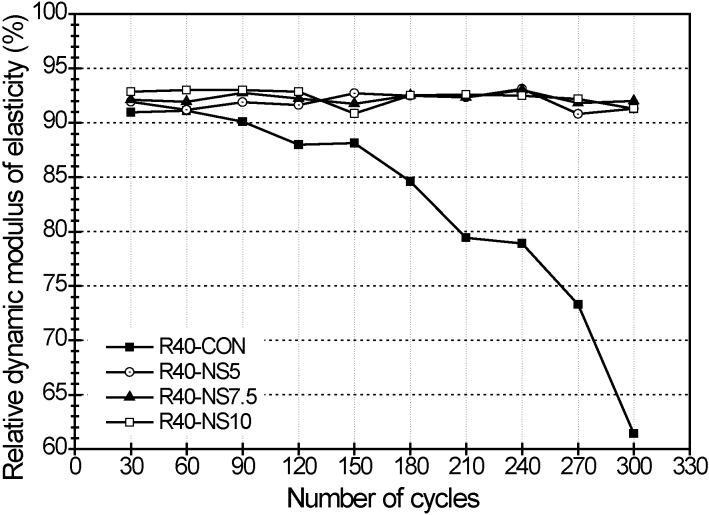
Relative dynamic modulus of elasticity by freezing-thawing cycles.

**Figure 5:**
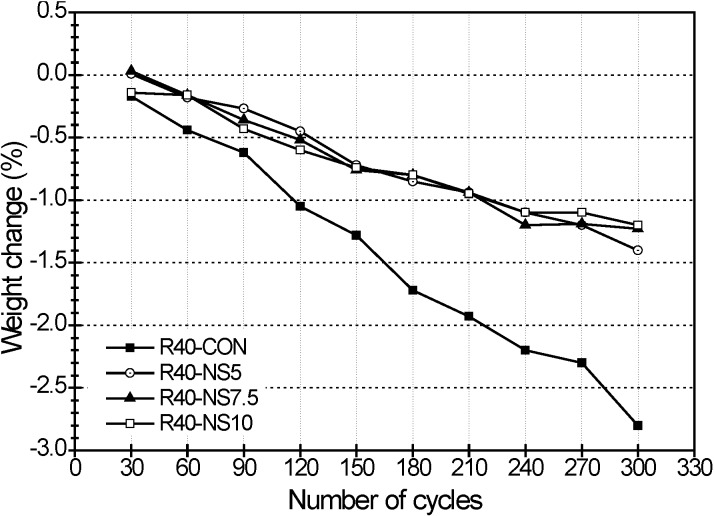
Weight change by freezing-thawing cycles.

The relative dynamic modulus of elasticity and weight change in the freezing and thawing test are presented in Figure 4 and Figure 5, respectively. The freezing and thawing test was carried out using the prism beam specimens with only W/C of 40%. The freezing and thawing test was performed for only admixtures with a W/C of 40% because of the capacity of equipment. After 30 cycles of lowering and raising the temperature, the relative dynamic modulus of elasticity became 91.0% in beam R40-CON, 91.9% in R40-NS5, 92.1% in R40-NS7.5, and 92.8% in R40-NS10, respectively. The relative dynamic modulus of elasticity changed to 88.1%, 92.7%, 91.8%, and 90.9% at 150 cycles. That in beam R40-CON with normal aggregate rapidly decreased after 150 cycles, and then it reached 61.4% at 300 cycles. However, the relative dynamic modulus of elasticity of the specimens with the MSCA barely changed while maintaining the range of 90%–95%.

The weight losses for each sample (e.g., R40-CON, R40-NS5, R40-NS7.5 and R40-NS10 prism beams) were 0.17%, 0.16%, 0.0% and 0.14% at 30 cycles, respectively. When 300 cycles passed, the weight losses in each sample were 2.8%, 1.4%, 1.23% and 1.2%. The loss of the control specimen was almost twice as large as that of the MSCA concrete. The weight losses of the MSCA concrete with different amounts of sulfur were not significantly different.

**Figure 6:**
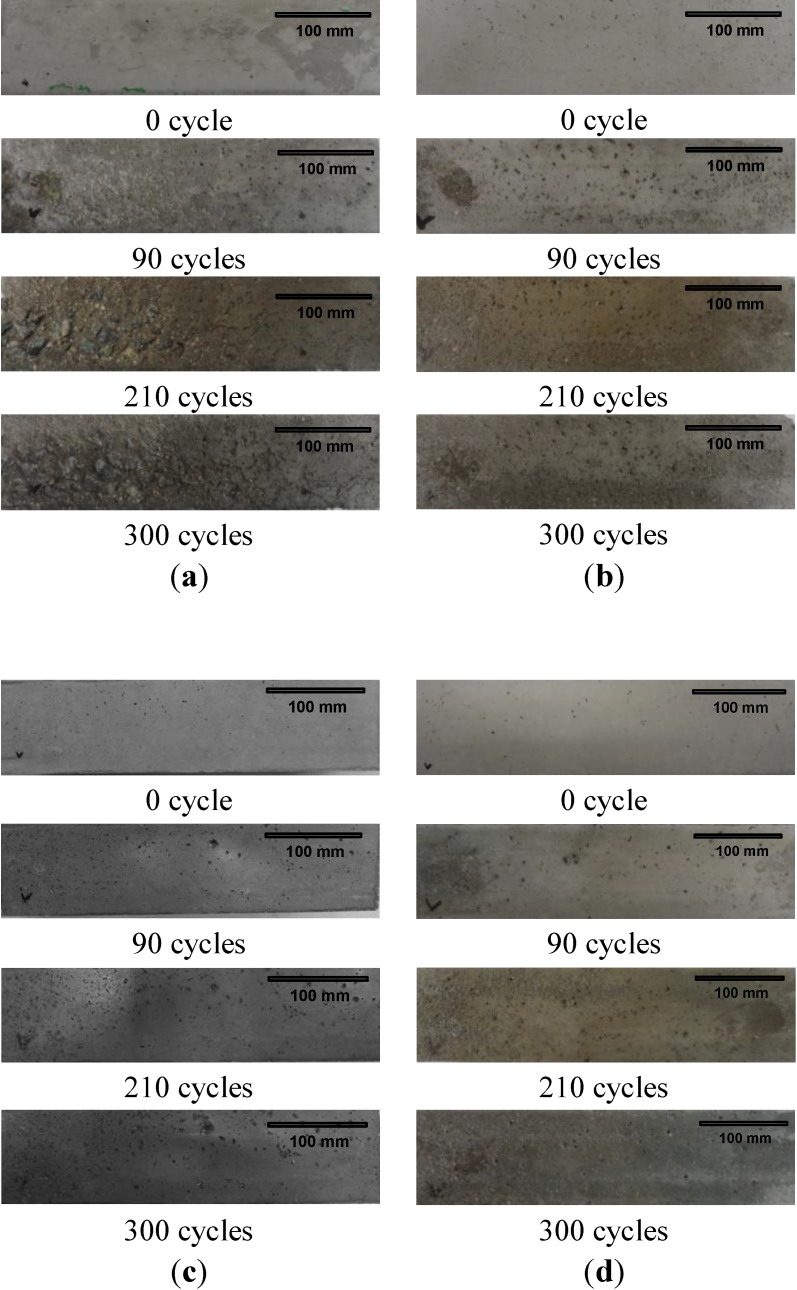
Surface status of (**a**) R40-CON; (**b**) R40-NS5; (**c**) R40-NS7.5; and (**d**) R40-NS10 specimens according to number of cycles.

Figure 6 shows the corrosion changes of four samples (e.g., R40-CON, R40-NS5, R40-NS7.5 and R40-NS10 prism beams) with passing cycles. 

The surface of the R40-CON sample started popping out considerably from 90 cycles, and exposure of the aggregate in the concrete was seen plainly after 180 cycles had passed. On half of the surface, aggregate exposure developed after 300 cycles, as illustrated in Figure 6a. In the MSCA concrete, the initial porosity faded after 300 cycles, but aggregate exposure due to popping out never occurred, as shown in Figure 6b–d. Based on the surface porosity observation, the MSCA significantly improved the freezing and thawing resistance. It was observed that the corrosion progress in R40-CON had been visually much more rapid than that in samples with MSCA. When water freezes, a volume expansion of 9.1% occurs, and the expansion moves to opening gap in cement paste. If there are no pores, mainly caused by entrained air, to relax the expansion, high pressure builds up in the concrete, and this is one cause of deterioration. Therefore, it was obvious that the use of MSCA results in the high porosity in the concrete and thus develops the resistance against the freezing and thawing of concrete.

### 2.6. Sulfate Resistance

**Figure 7:**
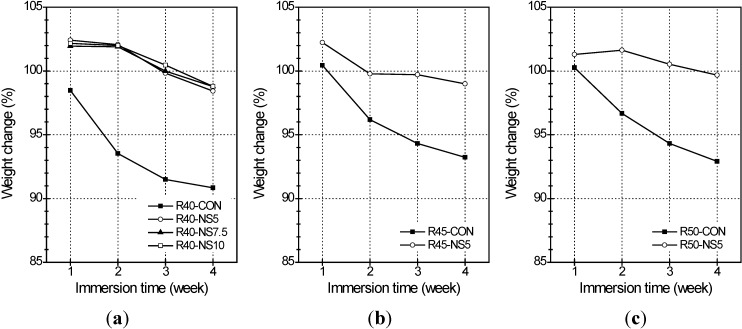
Weight change of cylinders with (**a**) W/C of 40%; (**b**) W/C of 45%; and (**c**) W/C of 50% in relation to W/C and immersion time.

The resistance of the MSCA concrete to sulfuric acid was investigated every 7 days for 4 weeks. The effect of MSCA on the sulfate attack resistance of concrete is shown in Figure 7. Of the samples with W/C = 40% (see Figure 7a), the weight in only RS40-CON with normal aggregate decreased by 1.5% after 1 week of immersion; however, that of the other samples with MSCA increased up to about 2% after 1 week. The weight increase may have been caused by absorption of the liquid phase due to concrete surface porosity. After that, the weight in all samples decreased gradually until 4 weeks. The final weight loss of the sample with normal aggregate was around 9% but those of the samples with MSCA were 1.2%–1.6%. There was little mass change, and it was insignificant for the samples with MSCA. It is known that the dilute hydrochloric and sulfuric acids, in general, cannot affect the sulfur. The sand and aggregate used in the concrete mixture consisted of mineral oxides of alkalinity. Acid damage on concrete is started from the chemical reactions of basic oxides, acid oxides, and amphoteric oxides. Because the coating modified sulfur prevents reaction between acid and basic oxides, the resistance to sulfate improves.

As compared to samples having MSCA with 5% modified sulfur, the weight losses were 1.2% in the W/C = 40%, 1.0% in the W/C = 45%, and 0.3% in the W/C = 50% samples after 4 weeks of immersion. Even though the loss of weight in two samples (R45-CON andR50-CON) with uncoated aggregate was not significantly different, the weight loss tended to be reduced with increased W/C. As test results, a higher W/C enhanced the acid resistance of the modified sulfur concrete.

**Figure 8:**
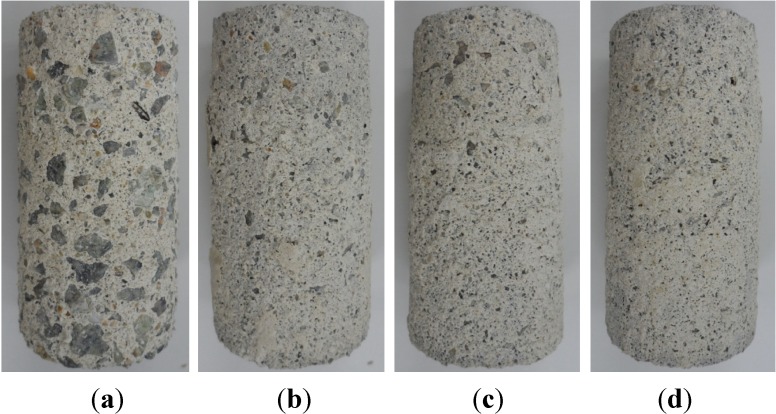
Surfaces of (**a**) R40-CON; (**b**) R40-NS5; (**c**) R40-NS7.5; and (**d**) R40-NS10 samples after 4 weeks of immersion in sulfuric acid.

Corrosion probably developed on the sample surfaces in open pores that were not coated or protected by sulfur and lasted until the test ended (see Figure 8). The specimen of R40-CON immersed in sulfuric acid solutions for 4 weeks showed high corrosion. By contrast, the specimens with MSCA showed few signs of corrosion. This indicates that MSCA contributes to better acid resistance in comparison with the control sample with normal aggregate.

## 3. Experimental Program

### 3.1. Materials

Type I Ordinary Portland Cement (OPC) conforming to the Korean standard was used in mixture designs, and the density and specific surface area of the cement were 3.15 g/cm^3^ and 3340 cm^2^/g, respectively. OPC has an average compressive strength of 50.2 MPa at a 28-day age. Its various chemical composition and physical properties are summarized in Table 1 and Table 2, respectively.

**Table 1:** Chemical composition of Type I Ordinary Portland Cement.

Chemical composition (%)								Loss Ignition
SiO_2_	CaO	Al_2_O_3_	Fe_2_O_3_	MgO	SO_3_	K_2_O	Na_2_O	
21.74	62.79	5	3.17	2.97	1.67	1.36	0.11	1.19

**Table 2:** Physical properties of Type I Ordinary Portland Cement.

Setting time (min)		Compressive strength (MPa)			Blaine specificsurface area (cm^2^/g)	Specific gravity
Initial Setting	Final Setting	@ 3-day	@ 7-day	@ 28-day		
221	303	30.32	41.50	50.20	3340	3.15

**Figure 9:**
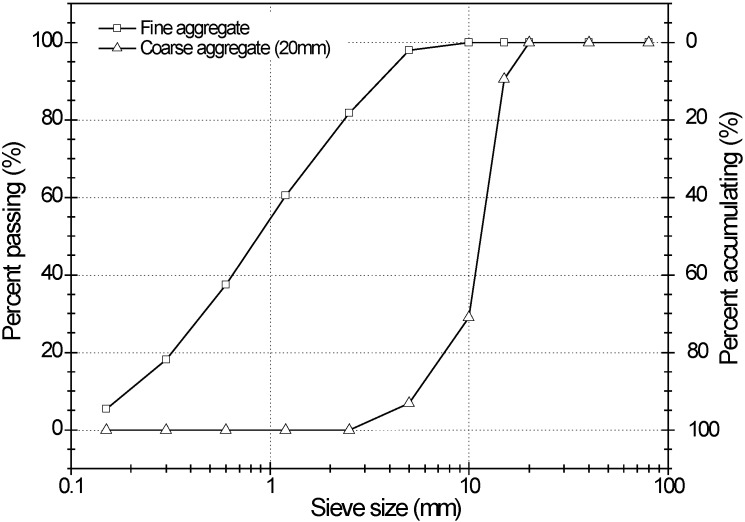
Grain size distribution of fine and coarse aggregate.

The physical properties of the modified sulfur, aggregates and superplasticizer used in this experiment were summarized in Table 3, Table 4 and Table 5. The modified sulfur was manufactured by polymerization with sulfur, dicyclopentadiene, and amine compound. The specific gravity was 1.75, the viscosity was 0.045 mPa·s at 85 °C, the polymerization degree was from 300 to 1200 g/mol, the melting temperature of sulfur was 80 °C, and the ratio of modified sulfur was 99.3% (Note that this ratio was measured by gel permeation chromatography, which is capable of measuring polymers from 500 to 10 million Daltons). The fine aggregate used in the concrete was natural river sand with a specific gravity of 2.60, a fineness modulus of 2.99, and water absorption of 2.90%. The granite coarse aggregate was crushed with a maximum particle size of 20 mm for the concrete mixture. Crushed aggregate has a specific gravity of 2.63, a fineness modulus of 7.03, and water absorption of 1.01%, which were measured according to ASTM C128 [23]. Figure 9 shows the grain size distribution of the aggregates used in the mix. Naphthalene superplasticizer was used to meet the target slump of 150 ± 25 mm. 

**Table 3:** Physical properties of modified sulfur.

Samples	Specific gravity	Viscosity (mPa·s)	Polymerization degree (g/mol)	Melting point (°C)	* Ratio of modified sulfur (%)
NS	1.75	0.045 at 85 °C	300–1200	80	99.3

* Ratio of modified sulfur was measured by using gel permeation chromatography.

**Table 4:** Physical properties of aggregates.

Type	Maximum size (mm)	Specific gravity	Fineness modulus	Water absorption (%)
Fine aggregate	–	2.6	2.99	2.9
Coarse aggregate	20	2.63	7.03	1.01

**Table 5:** Physical properties of superplasticizer.

Specific gravity (g/mL)	pH value	Temperature stability (°C)	Usage (%)
120 ± 0.2	7 ± 2	5–50	*C × (0.1–3.0)

* C is cement weight.

### 3.2. Mixture Proportion

The target slump and air content of concrete were considered to be 150 ± 25 mm and 5.0%, respectively. While the sand-aggregate ratio (S/a) of 45% and amount of water (W) of 185 kg/m^3^ were fixed, the variables are summarized as follows: (1) water-cement ratios (W/C) of 40%, 45%, and 50% and (2) MSCA-cement weight ratios of 0%, 5.0%, 7.5%, and 10.0%. Note that “R” in the mixture column represents the W/C, “CON” is the control mixture with uncoated crushed aggregate, “NS” is the modified sulfur used to coat the aggregate surface, and a final Arabic numeral is the modified sulfur-cement weight ratio. The detailed concrete mixing designs are given in Table 6. In R40 admixture series, 7.5% and 10% MACAs had negative effects on the mechanical properties of concrete, which made the coating treatment meaningless. So the mixtures with 7.5% and 10% MSCAs in R45 and R50 admixture series were not considered.

**Table 6:** Mix proportions of concrete mixtures.

Mixture	W/C (%)	S/a (%)	Air (%)	Unit weight (kg/m^3^)					Sulfur (C%)
				W	C	S	G	AD	
R40-CON	40	45	5	185	462	737	917	2.5	0.0
R40-NS5								4.3	5.0
R40-NS7.5								4.6	7.5
R40-NS10								4.9	10.0
R45-CON	45	45	5	185	411	756	942	2.2	0.0
R45-NS5								3.7	5.0
R50-CON	50	45	5	185	370	771	961	1.0	0.0
R50-NS5								3.1	5.0

W—water; C—cement; S—sand; a—aggregate; G—gravel; and AD—superplasticizer.

### 3.3. Specimen Preparation

**Figure 10:**
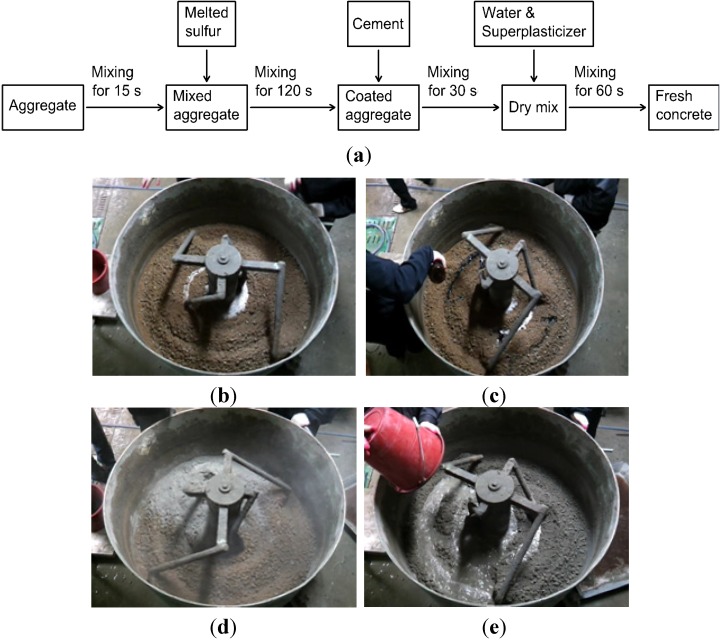
Concrete mixing method: (**a**) mixing procedures; (**b**) mixing of aggregate; (**c**) addition of modified sulfur; (**d**) dry mix; and (**e**) addition of water and superplasticizer.

Figure 10 illustrates the mixing procedure of the concrete with coated aggregate: (1) the coarse and fine aggregates were mixed for 15 s; (2) the melted sulfur was added and mixed to coat the aggregate surface at a speed of 20 rpm for 120 s; (3) the cement was added and dry-mixed with the coated aggregate for 30 s; and (4) finally, fresh concrete was made after mixing with the water and superplasticizer for 60 s. A fan-type mixer was used during mixing procedure. Concrete cylinders with dimensions of ϕ100 mm × 200 mm were made for the measurement of the compressive strength and sulfate resistance of the proposed concrete mix. Prism beam specimens with dimensions of 100 mm × 100 mm × 400 mm were also prepared for the flexural strength test, freezing-thawing and length change. The concrete cylinders and prism beams were cast in plastic and steel molds, respectively. After the concrete was poured in the molds, the molds were vibrated using the vibrating table. The specimens were wrapped with plastic sheeting to prevent moisture evaporation, and they were kept in the curing room at the temperature of 23 ± 2 °C and average relative humidity of 85%. When 24 ± 4 h passed, all specimens were demolded and water-cured until the various testing days.

**Figure 11:**
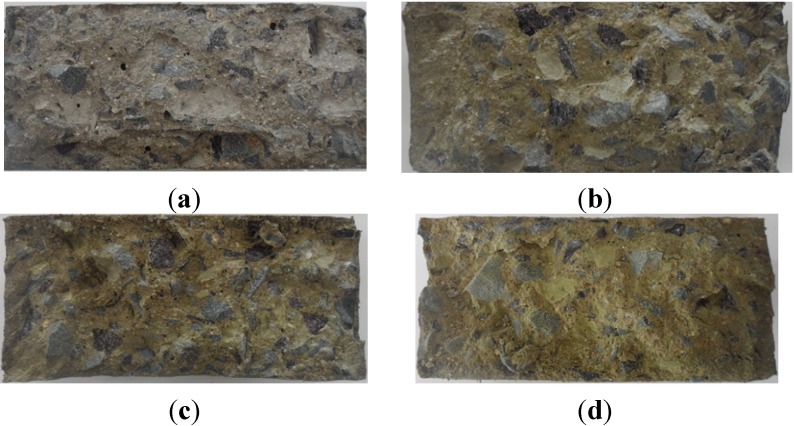
Fracture surfaces of (**a**) R40-CON; (**b**) R40-NS5; (**c**) R40-NS7.5; and (**d**) R40-NS10 cylinders after splitting test.

Distribution of sulfur on the surrounding aggregate was investigated by pictures taken after splitting failure, as shown in Figure 11. The modified sulfur was well-distributed all over the concrete and yellow color of concrete with MSCA became thicker with the higher amount of modified sulfur at the MSCA.

### 3.4. Tests

**Figure 12:**
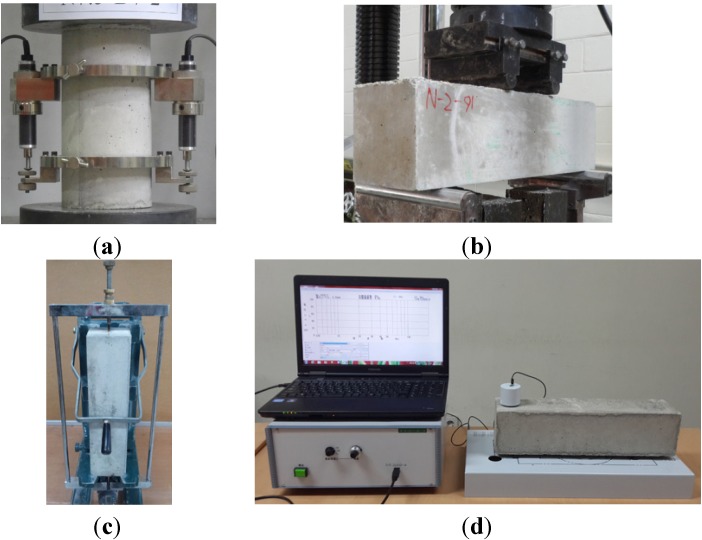
Test setup of (**a**) compressive strength; (**b**) flexural strength; (**c**) length change; and (**d**) dynamic modulus of elasticity.

The water absorption, total porosity, and bulk density of the hardened MSCA concrete were determined after 28 days of curing in accordance with requirement of ASTM C642 [24]. A compressive strength test was carried out according to the ASTM C39/C39M [25] at 7, 28, and 56 days after manufacturing the specimens. A universal test machine (UTM) with the capacity of 2000 kN was used for loading (see Figure 12a). The flexural strength was also measured according to the ASTM C78/C78M [26] using a load-cell with a range of 200 kN at 28 and 56 days (see Figure 12b). 

The length change of the prism specimen, as shown in Figure 12c, was evaluated by the comparator dial method in ASTM C157/C157M [27]. The data were recorded at 1 day intervals up to 7 days and continuously at 7 days intervals from 7 to 56 days. The resistance of the concrete to accelerate freezing and thawing in the prism specimens was carried out in accordance with ASTM C666/C666M [28]. The nominal freezing-and-thawing cycle for this test method consisted of alternately lowering the temperature of the specimens from 4 ± 2 to −18 ± 2 °C and raising it from −18 ± 2 to 4 ± 2 °C in 4 h. The weight and dynamic modulus of elasticity were measured at 30 cycle intervals for a total of 300 cycles. Figure 12d shows the test set up to measure the dynamic modulus of elasticity of a concrete beam after freezing-and-thawing cycles.

The sulfate attack resistance performance of the concrete was observed according to JSTM C7401 [29]. The concrete cylinder samples with dimensions of ϕ100 mm × 200 mm were deeply immersed in 5% H_2_SO_4_ solution at 20 ± 2 °C. The weight of the specimens before and after immersion was measured on a digital laboratory scale with a capacity of 5 kg and accuracy of 0.01 g every 7 days. The averaged data from three specimens of each mixture is used in next section of post-analysis and discussion.

## 4. Conclusions

In this paper, the use of aggregate coated with modified sulfur was proposed as a constructional material. Based on various experiments carried out using concrete specimens made with modified sulfur coated aggregate (MSCA), the following mechanical properties and durability were observed:

1. The greater the amount of modified sulfur used to coat the aggregate, the lower the compressive strength of the concrete was. This is because the modified sulfur remaining after coating can act as impurities or light-weight aggregate in the concrete. Five percent modified sulfur of MSCA barely affected the flexural strength of the concrete regardless of the water-cement ratio, while addition of more than 7.5% of modified sulfur led to quickly decreased strength.
2. Use of MSCA in concrete slightly reduced the length change of concrete as compared with concrete not having MSCA, but the change rates did not significantly differ with variation of the melted amount of sulfur in the MSCA.
3. Also, the MSCA considerably improved the resistance to freezing-thawing and sulfuric acid because its use resulted in high porosity and prevented a chemical reaction between acid and basic oxides. The dosage of sulfur, however, did not greatly affect the resistance.
4. Amounts of modified sulfur exceeding 5% had bad effects on mechanical strength through a compressive and flexural test. And the modified sulfur enhanced the durability in sulfuric acid and freezing-thawing, whereas the improvement did not strikingly vary according to the amount of modified sulfur. Synthetically, in terms of improvement of durability, using 5% modified sulfur was suggested restrictively in the MSCA concrete.
